# Smallholder Broiler Farmers' Characteristics to Uptake Measures Against Highly Pathogenic Avian Influenza in Western Java

**DOI:** 10.3389/fvets.2022.727006

**Published:** 2022-02-14

**Authors:** M. Gumilang Pramuwidyatama, Dikky Indrawan, Helmut W. Saatkamp, Henk Hogeveen

**Affiliations:** ^1^Business Economics Group, Wageningen University and Research, Wageningen, Netherlands; ^2^School of Business and Management, Bandung Institute of Technology, Bandung, Indonesia; ^3^School of Business, IPB University (Bogor Agricultural University), Bogor, Indonesia

**Keywords:** smallholder farmer, poultry, highly pathogenic avian influenza, prevention, control, business type, endemic

## Abstract

Highly Pathogenic Avian Influenza (HPAI) H5N1 remains endemic in the Western Java smallholder broiler farms. This study aims to identify farmers and farm characteristics associated with farmers' motivations toward five different measures directed to HPAI: cleaning and disinfection (C&D), vaccination, reporting, and stamping-out with and without compensation. Through multi-stage sampling and a questionnaire, we collected data from 199 farmers in Western Java and applied descriptive analysis and logistic regression to evaluate the data. Most smallholder broiler farms had a production contract with a poultry company. Unexpectedly, we identified subtypes of price-contract (i.e., revenues based on contract selling price and live bird weight) and *makloon*-contract (i.e., revenues based on management fee per bird) schemes. We identified these new subtypes as extended price-contract and extended *makloon-*contract schemes. These extended subtypes included issues related to animal health management and payment schemes. The results show that most of the farmers in both extended types were highly motivated to implement C&D and vaccination. Business types and farmers' awareness of HPAI were significantly associated with a farmer's motivation to implement C&D. Farmers who had an awareness of HPAI were more likely to implement C&D. Although our models were insufficient to model the association of farmers' motivation to uptake preventive measures against HPAI in Western Java, this study identified significant characteristics that help improve HPAI control policy in Western Java. Our study suggests that farm business types and incentives through payment schemes and training may increase the uptake of preventive measures by farmers.

## Introduction

Since the outbreak in 2004, HPAI has remained endemic in most of Indonesian regions. Efforts have been made to mitigate HPAI in Western Java, its control has not been completely successful. The broiler sector consists of a mix of industrialized, small-scale commercial, and backyard farms (1, 2), but the latter two account for more than 90% of the population and production (1). Due to the limited uptake of HPAI control measures by these types of broiler farmers (3), HPAI has become endemic in the region (4). Furthermore, there are markets for sick chickens in the traditional market chain, jeopardizing efforts to control HPAI in Western Java (1). Therefore, controlling HPAI at the farm level, particularly on small-scale commercial and backyard farms, is considered a priority strategy in the context of Western Java (5).

The three most common types of smallholder broiler farmers in Western Java are (a) independent, (b) *makloon*-contract, and (c) price-contract farmers (6). Independent farmers buy production inputs and sell chickens themselves. *Makloon*-contract farmers are paid by a large poultry company based on the number of day-old chickens at the start of the production cycle. Price-contract farmers have a contract with a larger poultry company to get production inputs and technical assistance on a credit basis and to sell their chickens to them at a predetermined price.

An analysis of determinants for smallholder broiler farmers' willingness to implement measures against HPAI in an endemic situation may inform the implementation of HPAI mitigation programs. To date, several studies have investigated the association between farmers' and farm characteristics and the uptake of preventive measures (7), biosecurity practices (6, 8), and vaccination programs (4). However, these studies focused on the uptake of measures aimed at disease prevention. For instance, Indrawan et al. (6) highlighted differences on overall farm biosecurity level and biosecurity infrastructures on different farm business types. In practice, a program usually comprises a set of different measures with different aims. This study compared factors that determine farmers' motivation to implement different measures aimed at prevention, monitoring, and control of HPAI. This way, the results of this study may be more informative for designing mitigation programs with a high adoption rate among farmers.

While the previous study (3) specifically focused on associations between socio-psychological factors and farmers' motivation, this study aims to identify if and how farmer and farm characteristics are associated with smallholder broiler farmers' motivation to implement different measures against HPAI, such as cleaning and disinfection (C&D), vaccination, reporting, and stamping-out.

## Materials and Methods

Since this study's materials and methods have been extensively presented in our published paper (3), a summary is presented in this section.

### Farm Level HPAI Mitigation Measures

This study investigates farmers' motivation to implement different preventive, monitoring, and control measures directed against HPAI, i.e., cleaning and disinfection (a proxy measure for biosecurity), vaccination, reporting, and stamping-out (3).

1. Cleaning and disinfection (C&D) is defined as cleaning and disinfecting the barn once in every 2 days.
2. Vaccination is defined as farmers vaccinate their flock against AI on the seventh day in every production cycle.
3. Reporting is defined as reporting to the technical service or veterinarian as quickly as possible if farmers observe AI infection symptoms on one of their chickens.
4. Stamping-out is defined as the culling of an entire flock after the farm has been declared infected by an HPAI virus. Two schemes exist: either farmers are compensated (up to 50% of the total value) for the culled healthy broilers, or they are not. The latter was the case in the current study.

### Questionnaire Design

The questionnaire used in this study was similar to a previously published version (3). The questionnaire comprised of two parts. The first part aimed to collect information about the farmer's sociodemographic characteristics, informational background, and farm business types. The second part aimed to collect information about intentions, attitudes, subjective norms, and perceived behavioral control related to the four mitigation measures mentioned above. In the current analysis, only information on farmer intentions was included.

In the first part on sociodemographic characteristics, parameters such as gender, age, education level, broiler farming contribution to farmers' income (i.e., the proportion of farmers' income received from broiler farming) were collected. The informational background of the farmers was also queried, through data on the poultry population, the farmer's broiler farming experience, and his/her knowledge about AI. To determine the farm's business type, farmers were asked whether they worked independently or had a production contract with an integrated company. Farmers with a production contract were subsequently asked whether they are paid based on the number of broilers on the farm (i.e., *makloon*-contract) or received payment based on total delivered weight (i.e., price-contract). We applied a checklist to identify parties responsible for providing production inputs, including day-old chicken (DOC), feed, veterinary drugs, vaccine, vitamin, and technical services (TS). Then we classified the checklist into farmers' autonomy, animal health management, production management, technical service assistance, financing, sales of chicken, production bonuses, and payment scheme.

In the second part, a five-point Likert scale was applied to measure farmers' agreement/disagreement (1 = strongly disagree, 2 = disagree, 3 = neutral, 4 = agree, 5 = strongly agree) to multiple motivation statements. We defined motivations using Ajzen's TACT principle, which stands for target, action, context, and time. For example, “If HPAI (target) were to occur in the environment where my farm is located (context) within one year (time), I would clean and disinfect the barn every 2 days (action).”

The questionnaire was written in English first, then translated to Bahasa Indonesia, and translated back to English for verification and publication. A pilot study with ten smallholder broiler farmers was done to test whether farmers could understand the statements. Afterward, some statements and terminologies were modified based on farmer input during the pilot study.

### Data Collection

The survey aimed to interview smallholder broiler farmers or staff responsible for the operations of the farm. Data were collected in four districts: Bogor, Subang, Tasikmalaya, and Ciamis regencies between March-April 2018. These districts were selected based on several criteria: population of broiler chickens, endemic HPAI, different dominant farming business types, and operational and logistical factors. Profiles of survey locations have been described by Pramuwidyatama et al. (3).

Stratified proportional (random) sampling was applied to have sufficient respondents from each business type. We also aimed to have 50 respondents from each regency. The stratification was based on personal communications with Indonesian poultry experts because there are no published data about the number of farms under different business types.

Two survey teams, each consisting of four enumerators, visited each regency on the same day. The team spent 2 days in each regency. Each team visited different subdistricts and was assisted by local government officials. Once teams arrived at the location, enumerators went to visit different farms individually to interview farmers. Snowball sampling was also applied to reach additional respondents by asking farmers or by approaching people who live nearby the farms.

In the end, we interviewed 223 farmers. Of those interviews, 20 were not finished because the farmer needed to leave during the interview. Four interviews with independent farmers were omitted from the analyses because the sample size was too small. Thus, 199 responses from price- and *makloon*-contract farmers were included in the analyses.

The study is exempted from ethics approval from the Social Sciences Ethics Committee of Wageningen University and Research (WUR). However, the survey complies with data collection and management rules in WUR and the codes of ethics for research involving human participants in Indonesia. These codes require that participants are well-informed about the aims of the research and anonymity in collecting and analyzing data (9).

### Data Analysis

First, the data were checked for errors and missing values. A descriptive analysis was applied to all variables used in this study. All the motivation variables' data turned out to be skewed, details are provided in Appendices 1, 2. Thus, logistic regression models were chosen to explain the associations between the characteristics and motivation (i.e., one model for each motivation). For all logistic regression models, farmers' motivations to implement each of the studied measures was used as dependent variables, and characteristics of farmers and farms were used as independent variables.

Each of the farmers' motivations was grouped into two categories based on the Likert scores given (i.e., 1–3: low or 4–5: high-level motivations) (3). Independent variables, such as gender, age, farming experience, poultry population, and awareness of HPAI and its signs, were also categorized as binary variables. Education and dependency level on broiler farming variables were categorized based on the number of choices used in each variable.

Univariable and multivariable logistic regressions were applied to analyze associations between dependent and independent variables. A Chi-square (χ^2^) test (or Fischer exact test when there were fewer than five responses in a cell) was applied to identify independent variables used in the logistic regression models. Independent variables with a *P* < 0.15 in either of the tests were included in the logistic regression models.

A backward stepwise procedure was applied to all multivariable logistic regression analyses. Independent variables that were not significant (*P* > 0.05) were removed from the models one-by-one at each step. All the statistical analyses were performed using IBM SPSS Statistics for Windows, version 25.0 (10).

## Results

### Contract Classification

During the fieldwork, we found subtypes of the price- and *makloon*-contract business types (Table 1). These subtypes included contract extensions related to animal health management decisions in both business types. In addition, a different payment scheme was found in *makloon*-contract business type.

**Table 1:** Classification of different business types in broiler farming based on the roles of broiler farmers and nucleus companies on different production and animal health aspects.

**Criteria**	**Business types**				
	**Independent**	**Price-contract**	**Extended price-contract**	**Extended *makloon*-contract**	***Makloon*** **-contract**
	Indrawan et.al. (6)	Indrawan et.al. (6)	(Study findings)	(Study findings)	Indrawan et.al. (6)
Farmers autonomy	Very high	High	Moderate	Low	Very low
Financing	Farmer	Farmer	Farmer	Nucleus	Nucleus
Production management	Farmer	Farmer	Farmer	Nucleus	Nucleus
Animal health management decisions^*^	Farmer	Farmer	Mostly farmer^*^	Mostly nucleus^*^	Nucleus
Production inputs (source of day-old chicken, feed, and animal health products)	Farmer	Nucleus	Nucleus	Nucleus	Nucleus
Technical service assistance	Animal health company	Nucleus/animal health company	Nucleus/animal health company	Nucleus/animal health company	Nucleus/animal health company
Production bonuses	No	Yes	Yes	Yes	Yes
Sales of chicken	Farmer	Nucleus	Nucleus	Nucleus	Nucleus
Payment scheme^*^	Live bird weight price based on market	Live bird weight price based on contract	Live bird weight price based on contract	Performance-based fee for each broiler sold^*^	Management fee for each day-old-chick reared

* *differences found in this study*.

We found that farmers in the price-contract type are not entirely in control of their flock's health. Instead, nucleus companies also control animal health management decisions, such as vaccination programs and disease control. As a result, these farmers had a lower level of autonomy in flock's health management. Thus, we categorized this business type as the extended price-contract type.

We also found that farmers in the *makloon*-contract type were allowed to make some adjustments to animal health management on their farms, instead of fully abiding management standards set by the nucleus company. In addition, the payment scheme of the *makloon*-contract business type was that the management fee paid to farmers per sold bird depends on the farm's performance instead of a fixed management fee based on the number of day-old-chicks. The higher the farm's performance in a production cycle, the higher *makloon* fee farmers will receive. Thus, these farmers had a higher level of autonomy in flock's health and production performance management. Based on this finding, we categorized this business type as the extended *makloon*-contract type.

This added another two business types to the three original business types (i.e., independent, price-contract, and *makloon*-contract) previously described by Indrawan et al. (6). The differences are shown in Table 2.

**Table 2:** Descriptive statistics of farmer and farm characteristics, and farmers motivations for each business type.

**Characteristics**	**Extended** ***makloon*****-contract** **(*****n*** **=** **141)**		**Extended price-contract** **(*****n*** **=** **58)**	
	***n***	**%**	***n***	**%**
**Geographical distribution**				
Bogor	36	26	9	15
Subang	22	16	29	50
Ciamis	53	38	–	–
Tasikmalaya	30	20	20	35
**Gender**				
Female	16	11	2	4
Male	125	89	56	96
**Age**				
<45 years old	56	40	41	71
≥45 years old	85	60	17	29
**Poultry population**				
≤ 3,000 chickens	82	58	25	43
>3,000 chickens	59	42	33	57
**Poultry farming experience**				
≤ 10 years	78	55	47	81
>10 years	63	45	11	19
**Age and farming experience**				
<45 years old and ≤ 10 years	40	28	35	60
<45 years old and >10 years	16	12	6	10
≥45 years old and ≤ 10 years	38	27	12	21
≥45 years old and >10 years	47	33	5	9
**Education**				
Elementary school	56	40	12	21
Junior high school	45	32	21	36
Senior high school and university	40	28	25	43
**Dependency level of broiler farming**				
>75%	75	54	29	57
50–75%	45	33	20	39
25–50%	18	13	2	4
<25%	–	–	–	–
**Awareness of HPAI and its signs**				
Yes	110	78	42	72
No	31	22	16	28
**High motivation toward:**				
Cleaning & disinfection		92		79
Vaccination		79		82
Reporting		89		86
Stamping-out without compensation		57		40
Stamping-out with 50% compensation		67		64

### Descriptive Statistics

Table 2 provides the descriptive statistics of business types, farmer characteristics, and farm characteristics based on interviews with smallholder broiler farmers. Of the 199 farmers interviewed and included in this study, 58 were extended price-contract farmers, and 141 were extended *makloon*- contract farmers. In contrast to our original expectations during sampling, we found more extended *makloon*-contract farmers in Bogor and Subang and no price-contract farmers in the Ciamis regency. Detailed results of the descriptive statistics are shown in Appendices 3, 4, respectively.

The majority of both extended *makloon*- and extended price-contract farmers were highly motivated to implement cleaning and disinfection (*makloon*: 92%; price: 79%), reporting (*makloon*: 89%; price: 86%), and vaccination (*makloon*: 79%; price: 82%). The motivation to apply stamping-out measures was clearly lower, both in the absence of compensation (*makloon*: 57%; price:40%) or with 50% compensation (*makloon*: 67%; price: 66%).

The large majority of smallholder broiler farmers were male. Most extended *makloon*-contract farmers were older than 45 years old, while most extended price-contract farmers were younger than 45. A higher proportion of the younger *makloon* farmers' had <10 years of farming experience, while the older group had a more or less a balance composition of farmers with shorter and longer farming experience. For price-contract farmers, most of the farmers had <10 years of farming experience in both age groups. On average, extended *makloon*-contract farmers had fewer than 3,000 birds on their farms, while extended price-contract farmers had more than 3,000 birds. There was also a marked difference in education level: extended *makloon*-contract farmers most often had elementary school education, while more extended price-contract farmers had education until senior high school. Both extended *makloon*- and price-contract farmers were highly dependent on their income from broiler farming; and had awareness of HPAI and its signs.

### Characteristics in Relation to Farmers' Motivations

Table 3 shows characteristics included and significantly associated with farmer motivation in each of the models. Detailed results of the univariable and multivariable logistic models are shown in Appendices 5, 6, respectively.

**Table 3:** Univariable and multivariable logistic regression model results showing significant associations between farm(er) characteristics and each prevention, monitoring, or control measure against HPAI.

**Characteristics**	**Cleaning & disinfection** **(*N* = 199)**	**Vaccination** **(*N* = 177)**	**Reporting** **(*N* = 199)**	**Stamping-out (no compensation)** **(*N* = 170)**	**Stamping-out (50% compensation)** **(*N* = 170)**
Business type	<0.05^a^, ^b^	-	-	<0.05^b^	-
Gender	-	-	-	<0.05^a^, ^b^	-
Age	-	-	-	-	-
Education	-	-	-	-	n.s.
Dependency level on broiler farming	-	n.s.	n.s.	-	-
Poultry population	-	-	<0.05^a^	n.s.	<0.01^a^, ^b^
Poultry farming experience	-	-	-	-	-
Awareness of HPAI and its signs	<0.05^a^, ^b^	-	n.s.	<0.01^a^; <0.05^b^	<0.01^a^; <0.05^b^
*R^2^*	0.102	0.059	0.064	0.112	0.133

a *Univariable model*;

b *Multivariable model; n.s., not significant; -, not used in the model based on Chi-square or Fisher's exact test (p > 0.15). Difference in the number of respondents is due to missing values*.

The business type was significantly (*P* < 0.05) associated with farmers' motivations toward cleaning and disinfection and stamping-out without any compensation. Extended price-contract farmers were less motivated to clean and disinfect (odds ratio, OR 0.34) or to join stamping-out without any compensation (OR 0.48).

Gender was significantly (*P* < 0.05) associated with farmers' motivation to join stamping-out without any compensation. Male farmers were more motivated than female farmers to join stamping-out in the absence of compensation.

The size of the poultry population was significantly and positively associated with farmer motivation toward reporting (*P* < 0.05; OR 2.7) and stamping-out with 50% compensation (*P* < 0.01; OR 2.98). Being aware of HPAI and its signs was significantly and positively associated with the motivations to clean and disinfect (*P* < 0.05; OR 2.78) and to join stamping-out with (*P* < 0.05; OR 2.23) and without (*P* < 0.05; OR 2.26) 50% compensation.

The only characteristic that was not significantly associated with the motivation to vaccinate, neither in the univariable nor multivariable model, was dependency level on broiler farming. Age and poultry farming experience were not included in any model based on the Chi-square or Fischer's exact test. Even though farmers' education and dependency level on broiler farming were included in the models toward vaccination, reporting, and stamping out with 50% compensation, these factors were not significantly associated with any of the motivations. Overall, all of the models had a relatively low *R*^2^ score of 0.133 or less.

## Discussion

The aim of this study was to understand which farmer and farm characteristics are associated with farmer motivation to implement different HPAI control measures. We assessed motivation for cleaning and disinfection (C&D), vaccination, reporting, and stamping-out with and without 50% compensation.

### Factors

#### Business Types

The first major finding of the study was the identification of differences in price-contract and *makloon*-contract business types. We identified these new business types as “extended,” as animal health management decisions were different than their non-extended counterparts. Instead of animal health management decisions were taken solely by extended price-contract farmers as reported by Indrawan et al. (6), we found that these farmers were required to join an animal health program, for instance, a vaccination program, set by the nucleus company. Furthermore, in the case of the extended *makloon*-contract business type, farmers were allowed to make decisions regarding chicken health as long as the decision was consulted with the technical adviser of the nucleus company beforehand. We also identified differences in payment schemes compared to the *makloon* business type as reported by Indrawan et al. (6): the extended *makloon*-contract farmers were paid based on performance and the number of chickens sent to slaughterhouses.

The observed differences in business types might be explained by the time of data collection of this study which was 2 years later than the data collection of Indrawan et al. (6). Since differences were associated with animal health management decisions and payment schemes, these differences could influence farmers' motivation to take up control measures. Therefore, we explicitly considered these differences during interviews with farmers and in this study's analysis and discussion.

#### Motivation

Our results show that farmers from both business types were more motivated to implement preventive measures (i.e., C&D and vaccination) compared to control measures (i.e., stamping-out with and without compensation), in line with local governmental priorities that prefer biosecurity and vaccination over stamping-out measures (11). Thus, it would be wise for policymakers to focus on preventive over control measures.

Multivariable models for preventive measures only showed associations between farm(er) characteristics and farmer motivations to implement cleaning and disinfection. For smallholder broiler farmers, this motivation was associated with the type of business and with the farmer's awareness of HPAI and its signs. In contrast, none of the farm(er) characteristics were associated with the farmer's motivation to vaccinate their chickens, suggesting that socio-psychological factors, such as attitude, subjective norm, and perceived behavioral control, were better factors to model a farmer's motivation to implement AI vaccination (3).

Extended *makloon*-contract farmers were more likely to be motivated to take up cleaning and disinfection than extended price-contract farmers. One possible explanation could be the different incentives offered in each business type. Financial incentives could increase farmers' willingness to adopt preventive measures (3, 5, 12).

In the extended *makloon*-contract business type, farmers have two types of incentives: the *makloon* fee and a performance bonus. Furthermore, expenses for all production inputs are covered by the company in *makloon* business types. Farmers are expected to manage the production cycle according to the standards set by the nucleus company. In contrast, the extended price-contract business type only offers farmers a performance bonus, and farmers need to pay for all production inputs. They would most probably be more willing to clean and disinfect their barn if it would affect their income.

#### Awareness of HPAI

Farmer awareness of HPAI and its clinical symptoms was significantly and positively associated with the motivation to clean and disinfect their barn. A majority of farmers from both business types were aware of HPAI and its signs. Ongoing communication, education and training can help to maintain farmer awareness (13). Information exchange about HPAI and related measures increased farm biosecurity uptake among Cambodian poultry farmers for the duration of the education program (14). In both business types, technical advisers have been suggested as proper communicators for farmers (3, 13).

### Policy Implications

The extended *makloon*-contract business types is promising for increasing the uptake of HPAI preventive measures within smallholder broiler farms, as this business type offers financial incentives: higher *makloon* fees and bonuses for farmers who perform above the standard. Furthermore, both vaccination and biosecurity standards and practices might have a higher likelihood of being uniformly implemented and enforced to all farmers at a lower cost in the extended *makloon*-contract business type since, in this business type, nucleus companies require farmers to abide their production standards and they are able to buy production inputs in a large quantity at a lower price than farmers.

As a policymaker and regulator, the government could facilitate a push strategy that provides incentives to increase all stakeholders' participation in HPAI control (5). Our study findings suggest that the government could apply different push strategies for different business types. For (extended) price-contract business type, the government needs to aim its policies at farmers directly. On the other hand, for (extended) *makloon*-contract business type, the government needs to target nucleus companies and their technical advisors. In practice, this means that local governments should identify the dominant business type in their district and adjust their strategy accordingly. Incentives from the government can be directed to complement existing incentives offered by nucleus companies. They could include, for instance, access to subsidized vaccines and the provision of trained vaccinators. The push strategy can be implemented through public-private partnership (PPP) and communication, information, and education programs (15).

### Limitations

Our study has several limitations, similar to what has been explained in our previous study, such as the inadequacy of the number of respondents from extended price-contract farmers; unavailability of disease or outbreak status of farms; and a general understanding of the factors that determine farmers' motivations (3). The finding that business type influences farmer motivation to uptake measures suggests that research design should consider characteristics of different business types, as not only this study shows, but other studies as well [e.g., (6, 16)]. The influence of the different business types might partly explain the low R-squared of models in this study, and socio-psychological factors were better factors to explain farmers motivation toward different HPAI preventive measures (3). However, we found some significant characteristics with *p*-value lower than 5 and 1% to improve HPAI policy in Western Java. Still, having five different measures in one study allowed us to identify similarities and differences in factors that influence farmer motivation for different measures.

## Conclusions

To conclude, smallholder broiler farmers are more motivated to implement preventive measures compared to control measures. The business type and farmers' awareness of HPAI were found to be associated with the farmer's motivation to clean and disinfect their barn. A push intervention, incentives, and communications between stakeholders were suggested to increase adoption and continued implementation of preventive measures. Further research is needed to evaluate farmers' willingness to join a contract type with different incentive schemes and animal health management programs.

## Data Availability Statement

The raw data supporting the conclusions of this article will be made available by the authors, without undue reservation.

## Ethics Statement

Ethical review and approval was not required for the study on human participants in accordance with the local legislation and institutional requirements. The patients/participants provided their written informed consent to participate in this study.

## Author Contributions

MP designed the study, collected and analyzed the data, and drafted the manuscript. DI, HS, and HH provided input on the design of the study, helped in interpreting the study results, and critically revised the manuscript. All authors contributed to the article and approved the submitted version.

## Funding

MP and DI are funded by an Indonesian Endowment Fund for Education (LPDP) scholarship.

## Conflict of Interest

The authors declare that the research was conducted in the absence of any commercial or financial relationships that could be construed as a potential conflict of interest.

## Publisher's Note

All claims expressed in this article are solely those of the authors and do not necessarily represent those of their affiliated organizations, or those of the publisher, the editors and the reviewers. Any product that may be evaluated in this article, or claim that may be made by its manufacturer, is not guaranteed or endorsed by the publisher.

## References

[1] Rich KM van Horne P Daryanto A Hogeveen H. Linking supply chain governance and biosecurity in the context of HPAI control in Western Java: a value chain perspective. Front Vet Sci. (2018) 5:5–7. 10.3389/fvets.2018.00094 29770325 PMC5940733

[2] Wibawa H Karo-Karo D Pribadi ES Bouma A Bodewes R Vernooij H. Exploring contacts facilitating transmission of influenza A (H5N1) virus between poultry farms in West Java, Indonesia: a major role for backyard farms? Prev Vet Med. (2018) 156:8–15. 10.1016/j.prevetmed.2018.04.008 29891149

[3] Pramuwidyatama MG Hogeveen H Saatkamp HW. Understanding the motivation of western java smallholder broiler farmers to uptake measures against highly pathogenic avian influenza (HPAI). Front Vet Sci. (2020) 7:362. 10.3389/fvets.2020.00362 32793640 PMC7394697

[4] Ilham N Iqbal M. Factors determining farmers' decision on highly pathogenic Avian Influenza vaccination at the small poultry farms in Western Java. Med Peternakan. (2011) 34:219. 10.5398/medpet.2011.34.3.219

[5] Indrawan D Stegeman A Daryanto Hogeveen AH. A push and pull intervention to control avian influenza: a lesson learned from the western java poultry sector. Jurnal Manajemen Agribisnis. (2019) 16:179. 10.17358/jma.16.3.179

[6] Indrawan D Cahyadi ER Daryanto A Hogeveen H. The role of farm business type on biosecurity practices in West Java broiler farms. Prev Vet Med. (2020) 176:104910. 10.1016/j.prevetmed.2020.104910 32032799

[7] Susilowati SH Patrick I Iqbal M Jubb T. The characteristics of the farm and the farmer that affect the adoption of biosecurity on smallholder poultry farms in Indonesia. Livestock Res Rural Dev. (2013) 25:582–8.

[8] Martindah E Ilham N Basuno E. Biosecurity level of poultry production cluster (PPC) in West Java, Indonesia. Int J Poultry Sci. (2014) 13:408–15. 10.3923/ijps.2014.408.415

[9] KNEPK. Pedoman Nasional Etik Penelitian Kesehatan. (2011). Available online at: http://www.ke.litbang.kemkes.go.id/kom14/wp-content/uploads/2017/12/Pedoman-Nasional-Etik-Penelitian-Kesehatan-2011-Unedited-Version.pdf (accessed December 20, 2019).

[10] IBM Corp. IBM SPSS Statistics for Windows, Version 25.0. Armonk, NY: IBM Corp (2017).

[11] Pramuwidyatama MG Hogeveen H Saatkamp HW. A systematic evaluation of measures against highly pathogenic avian influenza (HPAI) in Indonesia. Front Vet Sci. (2019) 6:33. 10.3389/fvets.2019.00033 30834252 PMC6387902

[12] Komaladara AAP Patrick I Hoang N. Contract bonus systems to encourage biosecurity adoption on small-scale broiler farms in Indonesia. Ani Prod Sci. (2018) 58:595–600. 10.1071/AN1584528948418

[13] Jayawinangun R Indrawan D Sutarman DC. How to communicate livestock hazard? An approach to improve farmers'risk preparedness. Jurnal Manajemen Agribisnis. (2019) 16:165. 10.17358/jma.16.3.165

[14] Bhandari DP Wollen TS Lohani MN. Preventing highly pathogenic avian influenza (HPAI) at the rural community level: a case study from Cambodia. Trop Ani Health Prod. (2011) 43:1071–3. 10.1007/s11250-011-9828-y 21442155

[15] Ministry of National Development Planning (MoNDP). National Strategic Plan For Avian Influenza Control and Pandemic Influenza Preparedness 2006–2008. Jakarta (2006). p. 1–75.

[16] Durr PA Wibowo MH Tarigan S Artanto S Rosyid MN Ignjatovic J. Defining “Sector 3” poultry layer farms in relation to H5N1-HPAI—an example from Java, Indonesia. Avian Dis. (2016) 60(Suppl. 1):183–90. 10.1637/11134-050815-Reg 27309054

