# Supplementary material for: Smallholder Broiler Farmers' Characteristics to Uptake Measures Against Highly Pathogenic Avian Influenza in Western Java
Source: Front Vet Sci. 2022 Feb 14;9:727006. doi: 10.3389/fvets.2022.727006 (PMC8883136; doi:10.3389/fvets.2022.727006)
Supplement: Supplementary file 1 [file Data_Sheet_1.docx]

Supplementary Material

Smallholder Broiler Farmers’ Characteristics to Uptake Measures against Highly Pathogenic Avian Influenza in Western Java

Muchammad Gumilang Pramuwidyatama^*^, Dikky Indrawan^*^, Helmut Willem Saatkamp, Henk Hogeveen

*** Correspondence:** Muchammad Gumilang Pramuwidyatama: [muchammad.gumilang@wur.nl](mailto:muchammad.gumilang@wur.nl); Dikky Indrawan: [rdikky@apps.ipb.ac.id](mailto:rdikky@apps.ipb.ac.id)

1. **Appendices**

**Appendix 1.** Distribution of responses of extended *makloon*-contract farmers on their motivation toward different measures directed against HPAI.

| Motivation | *N* | Strongly disagree | Disagree | Neutral | Agree | Strongly Agree |
| --- | --- | --- | --- | --- | --- | --- |
| Cleaning & disinfection | 141 | 0% | 1% | 6% | 57% | 35% |
| AI vaccination | 132 | 0% | 7% | 14% | 54% | 25% |
| Reporting | 141 | 0% | 4% | 7% | 53% | 36% |
| Stamping-out (no compensation) | 125 | 0% | 14% | 30% | 50% | 7% |
| Stamping-out (50% compensation) | 125 | 0% | 5% | 28% | 52% | 15% |

**Appendix 2.** Distribution of responses of extended price-contract farmers on their motivation toward different measures directed against HPAI.

| Motivation | *N* | Strongly disagree | Disagree | Neutral | Agree | Strongly Agree |
| --- | --- | --- | --- | --- | --- | --- |
| Cleaning & disinfection | 58 | 2% | 9% | 10% | 57% | 22% |
| AI vaccination | 45 | 0% | 4% | 13% | 56% | 27% |
| Reporting | 58 | 0% | 5% | 9% | 60% | 26% |
| Stamping-out (no compensation) | 45 | 9% | 29% | 22% | 38% | 2% |
| Stamping-out (50% compensation) | 45 | 2% | 13% | 20% | 58% | 7% |

**Appendix 3.** Descriptive statistics of farmer and farm characteristics of extended *price*-contract farms, the numbers show the number or percentage (in brackets) of farmers in each within-category who had high motivation to implement HPAI countermeasures. (N=199)

| Farmers’ characteristics |  | Extended Price-Contract (n=58) | | | |
| --- | --- | --- | --- | --- | --- |
|  | Cleaning & disinfection  (n=58) | Vaccination  (n=45) | Reporting (n=58) | Stamping-out (no compensation)  (n =45) | Stamping-out (with compensation)  (n=45) |
| Farmers’ with high motivation (*n*) | 46 (80) | 37 (82) | 50 (86) | 18 (31) | 29 (64) |
| Geographical distribution (*n*) |  |  |  |  |  |
| Bogor | 9 (100) | 7 (88) | **9 (100)*** | 3 (37.5) | 7 (88) |
| Subang | 24 (83) | 16 (84) | **29 (100)*** | 7 (36.8) | 14 (74) |
| Ciamis | - | - | **-** | - | - |
| Tasikmalaya | 13 (65) | 14 (78) | **12 (60)*** | 8 (44.4) | 8 (44) |
| Gender (%) |  |  |  |  |  |
| Female | 4.3 (100) | 5.4 (100) | 4 (100) | 5.6 (50) | **3.4 (50)*** |
| Male | 95.7 (79) | 94.6 (81) | 96 (86) | 94.4 (39.5) | **96.6 (65)*** |
| Age (%)^a^ |  |  |  |  |  |
| <45 years old | 67.4 (76) | 70.3 (84) | 70 (85) | **50 (29)*** | 55.2 (52) |
| ≥45 years old | 32.6 (88) | 29.7 (79) | 30 (88) | **50 (64.3)*** | 44.8 (93) |
| Education (%) |  |  |  |  |  |
| Elementary school | 21.7 (83) | 21.6 (89) | **24 (100)*** | **38.9 (77.8)*** | 24 (78) |
| Junior high school | 37 (81) | 29.7 (79) | **40 (95)*** | **27.8 (35.7)*** | 31 (64) |
| Senior high school and university | 41.3 (76) | 48.6 (82) | **36 (72)*** | **33.3 (27.3)*** | 45 (59) |
| Dependency level of broiler farming (%) |  |  |  |  |  |
| > 75% | 56.4 (76) | 54.5 (78) | 55.8 (83) | **33.3 (26.1)*** | **42.3 (48)*** |
| 50%-75% | 38.5 (75) | 39.4 (81) | 41.9 (90) | **55.6 (62.5)*** | **50 (81)*** |
| 25%-50% | 5.1 (100) | 6.1 (100) | 2.3 (50) | **11.1 (100)*** | **7.7 (100)*** |
| <25% | - | - | - |  | - |
| Poultry population (%) |  |  |  |  |  |
| ≤3000 | **34.8 (64)*** | 40.5 (75) | **34 (68)*** | 38.9 (35) | **31 (45)*** |
| >3000 | **65.2 (91)*** | 59.5 (88) | **66 (100)*** | 61.1 (44) | **69 (80)*** |
| Poultry farming experience (%) |  |  |  |  |  |
| ≤10 years | 76.1 (75) | 75.7 (78) | 80 (85) | 77.8 (38.9) | 76 (61) |
| >10 years | 23.9 (100) | 24.3 (100) | 20 (91) | 22.2 (44.4) | 24 (78) |
| Awareness of HPAI and its signs (%) |  |  |  |  |  |
| Yes | **80.4 (88)*** | 73 (84) | 76 (91) | 83.3 (47) | 79 (72) |
| No | **19.6 (56)*** | 27 (77) | 24 (75) | 16.7 (23) | 21 (46) |
| High motivation towards: |  |  |  |  |  |
| Cleaning & disinfection | - | 81 (81) | **86 (94)*** | 94.4 (45.9) | 86 (68) |
| Vaccination | 81 (81) | - | 81 (81) | 83.3 (40.5) | 79 (62) |
| Reporting | **93.5 (86)*** | 81 (81) | - |  | 90 (70) |
| Stamping-out (no compensation) |  |  |  | - |  |
| Stamping-out (50% compensation) | 67.6 (86) | 62 (79) | 70.3 (90) | **83.3 (51.7)*** | - |

Note: *Significant relationship at 5% level between the characteristic and the motivation of broiler farmers to implement the respective measures based on Chi-square or Fisher’s exact test.

Difference in the number of respondents is due to missing values.

**Appendix 4.** Descriptive statistics of farmer and farm characteristics of extended *makloon*-contract farms, the numbers show the number or percentage (in brackets) of farmers in each within-category who had high motivation to implement HPAI countermeasures. (N=199)

| Farmers’ characteristics |  | Extended *Makloon*-Contract (n=141) | | | | |
| --- | --- | --- | --- | --- | --- | --- |
|  | Cleaning & disinfection  (n=141) | | Vaccination  (n= 132) | Reporting  (n=141) | Stamping-out (no compensation)  (n =125) | Stamping-out (with compensation)  (n=125) |
| Farmers’ with high motivation (*n*) | 130 (92) | | 104 (79) | 126 (89) | 71 (50.4) | 84 (67) |
| Geographical distribution (*n*) |  | |  |  |  |  |
| Bogor | **31 (86)*** | | 28 (83) | 33 (92) | 19 (55.9) | 27 (80) |
| Subang | **18 (82)*** | | 16 (84) | 18 (82) | 12 (63.2) | 13 (68) |
| Ciamis | **53 (100)*** | | 42 (79) | 48 (91) | 28 (60.9) | 30 (65) |
| Tasikmalaya | **28 (93)*** | | 18 (69) | 27 (90) | 12 (46.2) | 14 (54) |
| Gender (%) |  | |  |  |  |  |
| Female | 11.5 (94) | | 11.5 (75) | 11 (88) | **4.2 (21.4)*** | 9.5 (57.1) |
| Male | 88.5 (92) | | 88.5 (79) | 89 (90) | **95.8 (61.3)*** | 90.5 (69) |
| Age (%)^a^ |  | |  |  |  |  |
| <45 years old | **43.1 (100)*** | | 41.3 (84) | 41.3 (93) | 40.8 (59.2) | 40.5 (69) |
| ≥45 years old | **56.9 (87)*** | | 58.7 (75) | 58.7 (87) | 59.2 (55.3) | 59.5 (66) |
| Education (%) |  | |  |  |  |  |
| Elementary school | 40 (93) | | 39.4 (77) | 40.5 (91) | 39.4 (58.3) | **42 (73)*** |
| Junior high school | 31.5 (91) | | 27.9 (73) | 31 (87) | 26.8 (48.7) | **24 (51)*** |
| Senior high school and university | 28.5 (93) | | 32.7 (87) | 28.6 (90) | 33.8 (63.2) | **34 (76)*** |
| Dependency level of broiler farming (%) |  | |  |  |  |  |
| > 75% | 55.9 (95) | | **61.8 (89)*** | **59.3 (97)*** | 14.5 (66.7) | **59.8 (73)*** |
| 50%-75% | 31.5 (89) | | **28.4 (66)*** | **28.5 (78)*** | 30.4 (47.7) | **26.8 (50)*** |
| 25%-50% | 12.6 (89) | | **9.8 (67)*** | **12.2 (83)*** | 55.1 (59.4) | **13.4 (77)*** |
| <25% | - | | - | - | **-** | - |
| Poultry population (%) |  | |  |  |  |  |
| ≤3000 | 60 (95) | | 55.8 (76) | 58 (89) | 49.3 (65.5) | **47.6 (57)*** |
| >3000 | 40 (88) | | 44.2 (82) | 42 (90) | 50.7 (65.5) | **52.4 (80)*** |
| Poultry farming experience (%) |  | |  |  |  |  |
| ≤10 years | 56.2 (94) | | 55.8 (82) | 55.6 (90) | 60.6 (63.2) | 57 (71) |
| >10 years | 44 (91) | | 44.2 (75) | 44.4 (89) | 39.4 (49.1) | 43 (63) |
| Awareness of HPAI and its signs (%) |  | |  |  |  |  |
| Yes | 78.5 (93) | | 78 (79) | 79.4 (91) | **84.5 (61.9)*** | **83.3 (72)*** |
| No | 21.5 (90) | | 22 (77) | 20.6 (84) | **15.5 (39.3)*** | **16.7 (50)*** |
| High motivation towards: |  | |  |  |  |  |
| Cleaning & disinfection | - | | **95.2 (81)*** | 93.7 (91) | **98.6 (60.9)*** | 91 (68) |
| Vaccination | **81 (95)*** | | - | 81.2 (91) | 83.1 (60.8) | 81 (70) |
| Reporting | 91 (94) | | 91.3 (81) | - | 87.3 (56.4) | 85.7 (66) |
| Stamping-out (no compensation) |  | |  |  | - |  |
| Stamping-out (50% compensation) | 67.8 (93) | | 70.1 (81) | 65.5 (86) | **87.3 (73.8)*** | - |

Note: *Significant relationship at 5% level between the characteristic and the motivation of broiler farmers to implement the respective measures based on Chi-square or Fisher’s exact test.

Difference in the number of respondents is due to missing values.

**Appendix 5.** Univariable model results showing the odds ratio and 95% confidence interval (in a bracket) of the association of farmers’ characteristics with farmers’ motivation to implement routine cleaning and disinfection of the farm, HPAI vaccination, report an outbreak and to join stamping-out

| Farmers’ characteristics | Cleaning & disinfection  (*N*=189) | Vaccination  (*N*=171) | Reporting  (*N*= 189) | Stamping-out: no compensation  (*N*= 164) | Stamping-out: 50% compensation  (*N*= 164) |
| --- | --- | --- | --- | --- | --- |
| Farming scheme |  |  |  |  |  |
| Extended *Makloon*-Contract | 1.00 |  |  | 1.00 |  |
| Extended Price-Contract | 0.3 (0.13-0.79)* |  |  | 0.5 (0.25-1.01) |  |
| Gender |  |  |  |  |  |
| Female |  |  |  | 1.00 |  |
| Male |  |  |  | 3.69 (1.14-11.97)* |  |
| Education (N=199) |  |  |  |  |  |
| Elementary school |  |  |  |  | 1.00 |
| Junior high school |  |  |  |  | 0.43 (0.19-0.96)* |
| Senior high school & university |  |  |  |  | 0.83 (0.37-1.87) |
| Dependency level of broiler farming |  |  |  |  |  |
| 25%-50% |  | 1.00 | 1.00 |  |  |
| 50%-75% |  | 0.97 (0.3-3.2) | 1.1 (0.3-3.9) |  |  |
| > 75% |  | 2.6 (0.79-8.6) | 3.5 (0.9-13.2) |  |  |
| Poultry population |  |  |  |  |  |
| ≤ 3000 birds |  |  | 1.00 | 1.00 | 1.00 |
| > 3000 birds |  |  | 2.7 (1.02-7.2)* | 1.63 (0.89-2.99) | 3.35 (1.68-6.65)** |
| Awareness of HPAI and its signs |  |  |  |  |  |
| No | 1.00 |  | 1.00 | 1.00 | 1.00 |
| Yes | 2.9 (1.12-7.1)* |  | 2.3 (0.94-5.8) | 2.7 (1.3-5.6)** | 2.71 (1.32-5.6)** |

Statistics =" *significant at p<0.05, ** significant at p<0.01"

Difference in the number of respondents is due to missing values.

**Appendix 6.** Multivariable model results showing the odds ratio and 95% confidence interval (in a bracket) of characteristics that were retained in the farmers’ motivation models related to measures against HPAI.

| Farmers’ characteristics | Cleaning & disinfection  (*N*=189) | Vaccination  (*N*=171) | Reporting  (*N*= 189) | Stamping-out: no compensation  (*N*= 164) | Stamping-out: 50% compensation  (*N*= 164) |
| --- | --- | --- | --- | --- | --- |
| Farming scheme |  |  |  |  |  |
| Extended *Makloon*-Contract | 1.00 |  |  | 1.00 |  |
| Extended Price-Contract | 0.34 (0.14-0.82)* |  |  | 0.5 (0.24-0.99)* |  |
| Gender |  |  |  |  |  |
| Female |  |  |  | 1.00 |  |
| Male |  |  |  | 3.41 (1.01-11.45)* |  |
| Education (N=199) |  |  |  |  |  |
| Elementary school |  |  |  |  |  |
| Junior high school |  |  |  |  |  |
| Senior high school & university |  |  |  |  |  |
| Dependency level of broiler farming |  |  |  |  |  |
| 25%-50% |  | 1.00 | 1.00 |  |  |
| 50%-75% |  | 0.97 (0.3-3.2) | 1.1 (0.3-3.9) |  |  |
| > 75% |  | 2.6 (0.79-8.6) | 3.5 (0.9-13.2) |  |  |
| Poultry population |  |  |  |  |  |
| ≤ 3000 birds |  |  |  |  | 1.00 |
| > 3000 birds |  |  |  |  | 2.98 (1.48-6)** |
| Awareness of HPAI and its signs |  |  |  |  |  |
| No | 1.00 |  |  | 1.00 | 1.00 |
| Yes | 2.8 (1.12-6.97)* |  |  | 2.26 (1.06-4.84)* | 2.23 (1.05-4.72)** |
| ***R^2^*** | 0.102 | 0.059 | 0.064 | 0.112 | 0.133 |

Statistics =" *significant at p<0.05, ** significant at p<0.01"

Difference in the number of respondents is due to missing values.
